# Shen Shuai IIRecipe attenuates renal injury and fibrosis in chronic kidney disease by regulating NLRP3 inflammasome and Sirt1/Smad3 deacetylation pathway

**DOI:** 10.1186/s12906-019-2524-6

**Published:** 2019-05-22

**Authors:** Meng Wang, Liuyi Yang, Jing Yang, Chen Wang

**Affiliations:** 1 Department of Nephrology, Shuguang Hospital Affiliated to Shanghai University of Traditional Chinese Medicine, Shanghai, 201203 China; 2 Key Laboratory of Liver and Kidney Diseases,Ministry of Education, Shanghai University of Traditional Chinese Medicine, Shanghai, 201203 China; 3 TCM institute of kidney disease, Shanghai University of Traditional Chinese Medicine, Shanghai, 201203 China; 4 Shanghai Key Laboratory of Traditional Chinese Clinical Medicine, Shanghai University of Traditional Chinese Medicine, Shanghai, 201203 China

**Keywords:** Shen Shuai IIRecipe,Chronic kidney disease, NLRP3 inflammasome, Renal fibrosis, Deacetylation

## Abstract

**Background:**

Excessive activation of NLRP3 inflammasome and down-regulation of Sirt1/Smad3 deacetylation pathway play a significant role in the evolution of renal fibrosis. In China, it has been well known that Chinese herbal medicine is markedly effective in treating chronic kidney disease (CKD). Shen Shuai IIRecipe (SSR) has been used clinically for more than 20 years and has been confirmed to be effective in improvements of renal function and fibrosis. However, the specific mechanisms under the efficacy require further research. The purpose of this study was to evaluate whether SSR could alleviate renal injury and fibrosis by regulating NLRP3 inflammasome and Sirt1/Smad3 deacetylation pathway.

**Methods:**

Four weeks after 5/6 ablation/infarction (A/I) surgery, Sprague-Dawley rats were randomly divided into the following groups: sham operation group, 5/6 (A/I) group, 5/6 (A/I) + SSR group, and 5/6 (A/I) + Losartan group (5/6 (A/I) + Los). After 8 weeks intervention,we mainly assessed the severity of renal injury and fibrosis along with the activation of NLRP3 inflammasome and Sirt1/Smad3 deacetylation pathway.

**Results:**

SSR significantly attenuated renal injury and fibrosis in the remnant kidneys. In addition, we found that SSR effectively inhibited activation of NLRP3/ASC/Caspase-1/IL-1βcascade, decreased inflammatory infiltration and up-regulated Sirt1/Smad3 deacetylation pathway.

**Conclusions:**

SSR could contribute to renal protection by inhibiting the activation of NLRP3 inflammasome and, furthermore, strengthen the antifibrotic effects by up-regulating Sirt1/Smad3 deacetylation pathway in 5/6 renal (A/I) model.

## Background

Chronic kidney disease (CKD) affects over 10% of the human population worldwide with high mortality, in part due to limited available or affordable treatment [1]. The prevalence of CKD is increasing worldwide and renal fibrosis is the common outcome of CKD, irrespective of the initial causes [2, 3]. The activation of renal fibroblasts and excessive accumulation of extracellular matrix (ECM) components are the hallmark of renal tubulointerstitial fibrosis,which is closely interconnected with tissue regeneration and inflammation [4].

Tubulointerstitial inflammation is a critical pathological feature of CKD and promotes the evolution of interstitial fibrosis [5].NLRP3,a NOD-like pattern recognition receptor,initiates innate immune response during tissue injury or pathogen infections [6]. Upon activation,the NLRP3 proteins undergo oligomerization and recruit the adaptor protein ASC and pro-caspase-1 to form inflammasome [7, 8]. The formation of the inflammasome facilitates the release of active caspase-1 and the subsequent secretion of mature pro-inflammatory cytokines interleukin (IL)-1β and IL-18.Previous reports suggested the NLRP3 complex could play a significant role in promoting inflammatory response and renal fibrosis in CKD [9]. Zhuang et al. [10] demonstrated activation of NLRP3 inflammasome induced by proteinuria was involved in the progressive loss of renal function in CKD. Ludwig-Portugall et al. [11]. confirmed that CP-456,773,a specific inhibitor of the NLRP3 inflammasome, attenuated crytal-induced kidney fibrosis.

Transforming growth factorβ-1 (TGFβ-1),a profibrotic factor, has been shown to implicate in activation of renal fibroblasts and accumulation of ECM proteins [12].TGF-β1 signal activates Smad2 and Smad3 phosphorylation and subsequently forms a complex with Smad4 to modulate the transcription of target genes [13, 14].However,previous studies reported acetylation of Smad3 was involved in TGFβ1-dependent renal fibrosis [15]. Sirtuin 1(Sirt1),a conserved NAD^+^-dependent protein deacetylase, is a survival factor that is involved in lifespan extension and has been demonstrated to deacetylate the lysine residues [16]. He et al. [17].confirmed that activation of Sirt1 attenuated kidney fibrosis in UUO model. Therefore, the inhibition of NLRP3 inflammasome and activation of Sirt1 could be attractive targets to ameliorate renal fibrosis.

Traditional Chinese medicine (TCM) has been widely used for disease prevention and treatment over many years of clinical validation and become popular for promoting healthcare [18]. In China, it has been well known that Chinese herbal medicine is markedly effective in treating CKD. Shen Shuai IIRecipe (SSR),an effective prescription for the treatment of CKD in the clinic, is composed of Epimedium,Codonopsis pilosula, Salvia miltiorrhiza bge, *Rheum palmatum*, Angelica sinensis, Folium perillae, Coptis chinensis, Peach kernel,and Ligusticum chuanxiong, aiming to‘fortify the spleen and tonify the kidney’and‘activate blood and resolve stasis’.Clinically, SSR has been used for more than 20 years and become the basic prescription for the treatment of patients with CKD. Previous clinical studies suggested SSR significantly improved renal function of patients with CKD, as evidenced by decreased levels of serum creatinine (Scr),24-h urinary protein quantity, blood urea nitrogen (BUN) and increased evaluated glomerular filtration rates (eGFRs) [19]. Renal ultrasound detection revealed that SSR markedly increased renal blood flow in primary CKD 3 or 4 stage patients [19]. Previous experimental studies showed that SSR significantly down-regulated the expression of NF-κB/TNF-α signaling pathway and attenuated renal fibrosis by improving oxygen consumption in 5/6 renal ablation/infarction (A/I) model [20, 21]. Importantly, We didn’t observe any side effects or changes of liver function in clinical patients and experimental animals. These findings may indicate that the therapeutic effect of SSR is devoid of any toxic manifestation**.** In the present study, we aimed to investigate whether SSR could alleviate renal fibrosis by regulating NLRP3 inflammasome and Sirt1/Smad3 deacetylation pathway.

## Methods

### Animals and drugs

Eight-week-old male Sprague-Dawley (SD) rats (190 g–210 g) were obtained from SLAC Laboratory Animal Co., Ltd. (Shanghai,China) and maintained under a controlled temperature (23 ± 3 °C) and humidity (55 ± 15%) with a 12 h light/12 h dark cycle. SSR consists of Epimedium 15 g,Codonopsis pilosula 15 g,Salvia miltiorrhiza bge 15 g,*Rheum palmatum* 15 g,Angelica sinensis 15 g,Folium perillae 15 g,Coptis chinensis 6 g,Peach kernel 15 g,and Ligusticum chuanxiong 15 g. The raw herbs for preparation of SSR were obtained from Shanghai Kangqiao Chinese Medicine Tablet Co.,Ltd. (Shanghai,China) and identified by Dr. Guanglin Xu from Pharmacy Department, Shuguang Hospital Affiliated to Shanghai University of TCM. The above nine commonly used Chinese herbs were mixed with distilled water in proportion and heated twice at 100 °C for 1 h under continuous stirring condition. The aqueous extracts were subjected to centrifugation at 1500×g. The supernatants were merged and concentrated to prepare suspension containing 6 g/mL of the original drug by means of ethanol. The gavage dose (10 mL/kg) in the present study was determined according to the previous studies [21]. Losartan tablets (100 mg/tablet) were obtained from MSD Pharmaceutical Co., Ltd.(Hangzhou,China) and used as positive control. Losartan was dissolved and diluted by saline with a concentration of 5 mg/mL of solution.

### Rat 5/6(A/I) model and animal study protocol

5/6 (A/I) surgery was performed as described previously [21, 22]. The rats were anesthetized with sodium pentobarbital (40 mg/kg, i.p.), and placed on temperature-controlled surgical table. The surgical area was disinfected with 75% alcohol and a flank incision was then performed. The left kidney was taken out from the retroperitoneum and the renal artery of left kidney was exposed after separation of perirenal fat and renal capsular. Two branches of the left renal artery were ligated with 4–0 silk sutures and the gelatin sponge was used to suppress the bleeding for a moment. The left kidney was gently retracted back into the body and the incision was sutured. After one week,a right flank incision was performed. After separating the adrenal gland, the right renal pedicle was ligated and the kidney was removed. 45 rats were randomized into three groups after 4 weeks and administered SSR (10 mL/kg daily by gavage,*n* = 15),losartan (6 mL/kg daily by gavage,n = 15) or saline treatment. The sham group underwent the same anesthesia and procedures consisting of manipulation of the perirenal fat and renal pedicles, except for the destruction of renal tissue. A group of 15 rats that received sham operation were also included in the study. After 8 weeks intervention,all animals were anaesthetized with sodium pentobarbital (40 mg/kg) administered intraperitoneally and euthanized by cervical dislocation. The remnant kidney tissues were collected for molecular examination. The animal procedures were approved by the Animal Experiment Ethics Committee of Shanghai University of Traditional Chinese Medicine in accordance with the principles outlined in the NIH Guide for the Care and Use of Laboratory Animals.

### Immunoprecipitation analysis

Kidney tissues were lysed on ice for 15 min in lysis buffer containing protease inhibitor. Approximately 500 μg of total protein was incubated overnight at 4 °C with anti-Smad3(Santa Cruz,USA) followed by precipitation with 70 μl of protein A/G-Plus-Agarose (Santa Cruz,USA) for 4 h at 4 °C.Non-specific IgG (Proteintech) was used as negative control. The precipitated complexes were washed in IP buffer and then, resuspended in 30 μl of 2× loading buffer and boiled for 5 min.

### Western blot

The proteins were isolated from the frozen kidneys and the concentration was calculated by the Bradford method. Immunoblotting examination was performed as previously described [21]. In this study, the primary antibodies used were anti-Ecadherin (1:1000,Abcam,UK), anti-ColIII (11,000, Abcam, UK),anti-caspase1. (1:1000, Abcam,UK), anti-TGFβR1 (1:1000,Abcam,UK), anti-Sirt1 (11,000, Abcam, UK), anti-NLRP3 (1:1000, Abclonal, China), anti-IL-1β (11,000,Abclonal,China), anti-Acetylated-lysine (1:500,Santa Cruz,USA), anti-ASC (1:500, Santa Cruz, USA), anti-TGF-β1 (1500,Santa Cruz,USA), anti-Smad3 (1,1000,CST,USA), and anti-Gapdh (12,000, Proteintech, USA). Protein bands were visualized using the chemiluminescence system (Tanon) for the required time. Quantitative analysis was performed using ImageJ software.

### Histology staining and evaluation

Paraffin-embedded kidneys were sectioned into 3 μm slices and subjected to Masson’s trichrome and periodic acid-Schiff (PAS) staining according to the standard protocol. For PAS-stained sections,the severity of glomerular injury was assessed in 50 glomeruli using the glomerulosclerosis (GS) score from 0 to 4 and the mean score was calculated [23]. The severity of tubulointerstitial fibrosis was calculated by the Masson’s trichrome-positive areas in 4 randomly selected fields per section of kidney using Image-Pro Plus 6.0.

### Immunohistochemical (IHC) and immunofluorescence (IF) staining

IHC staining was performed as described previously [21]. In this study, the primary antibodies used were anti-Ecadherin (1200,Abcam) and anti-Sirt1 (1200, Abcam). Analysis of semi-quantification was performed in 4 randomly selected fields per section of kidney using Image-Pro Plus 6.0.For immunofluorescence staining,antigen was retrieved by microwave-EDTA buffer antigen retrieval method and blocked with 3% BSA and 0.1% Triton-100 after quenching autofluorescence. The sections were incubated with anti-αSMA (1200,Abcam,UK) and anti-F4/80(1200,Servicebio, China) overnight at 4 °C.The following secondary antibodies were used: FITC-labeled goat anti-rabbit IgG (Beyotime,China) and Cy3-labeled goat anti-mouse IgG (Beyotime,China). Positive staining was observed by fluorescence microscopy (Nikon Eclipse 80i, Japan) at 400 × magnification.

### Statistical analysis

All results were expressed as mean ± standard deviation (SD) and analyzed by one-way analysis of variance (ANOVA) with LSD-t’s multiple comparisons, using SPSS version 18.0 software. *P* < 0.05 was considered statistically significant.

## Results

### SSR attenuated renal injury and fibrosis in 5/6(A/I) model

**Fig. 1:**
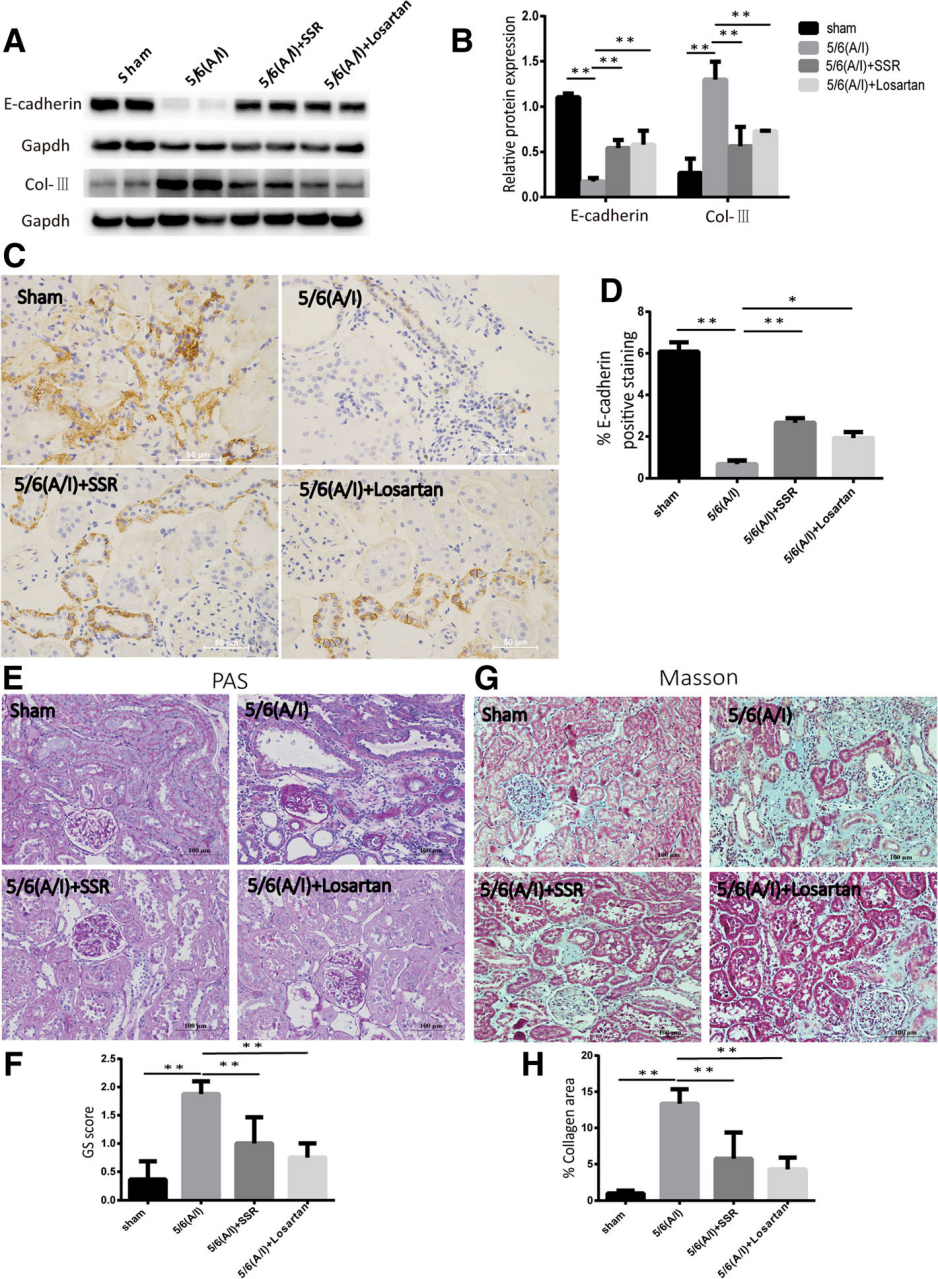
SSR attenuated renal injury and fibrosis in 5/6(A/I) model. (**a**) Immunoblot analysis of E-cadherin and Col-IIIexpression. (**b**) Western blot quantification of E-cadherin and Col-III levels.(*n* = 6) (**c**) Representative images of E-cadherin expression detected by IHC. 400 × magnification. (**d**) Quantitative analysis of E-cadherin positive area(*n* = 4) (**e**) Representative photomicrographs of PAS staining. 200 × magnification. (**f**) The severity of glomerular injury was assessed using the glomerulosclerosis (GS) score (**g**) Assessment of interstitial fibrosis by Masson’s trichrome staining.200 × magnification. (**h**) Semiquantitative result of collagen area(n = 4). Values are mean ± SD. *P < 0.05, **P < 0.01

In previous studies,we confirmed SSR significantly attenuated the expression of Collagen-I (Col-I) and fibronectin (FN) proteins,two major ECM proteins, as well as α-smooth muscle actin (α-SMA) protein,the hallmark of fibroblast activation [21]. Based on the previous results, the study aimed to further assesse the expression of Collagen-III (Col-III) and E-cadherin proteins. Immunoblotting examination showed the expression of Col-III and loss of the epithelial cell marker E-cadherin were increased in the remnant kidneys (Fig. 1(a) and (b)).The IHC analysis of E-cadherin expression showed the same pattern as that of immunoblotting in 5/6(A/I) group (Fig. 1(c) and (d)). Conversely,SSR treatment markedly increased E-cadherin expression and decreased Col-III expression at protein levels in 5/6 (A/I) rats (Fig. 1(a),(b),(c) and(d)).

Consistent with above observations, histopathological examination confirmed SSR showed significant reduction in interstitial fibrosis and renal injury when compared with model group (Fig. 1(e),(f),(g)and1(h)).SSR treatment for 8 weeks displayed less tubular injury, tubulointerstitial inflammation,and fibrosis after 5/6(A/I) operation,verifying a protective effect for SSR in renal injury (Fig. 1(e),(f), (g)and1(h)).

SSR inhibited NLRP3 inflammasome activation and IL-1β secretion in 5/6(A/I) rats.

**Fig. 2:**
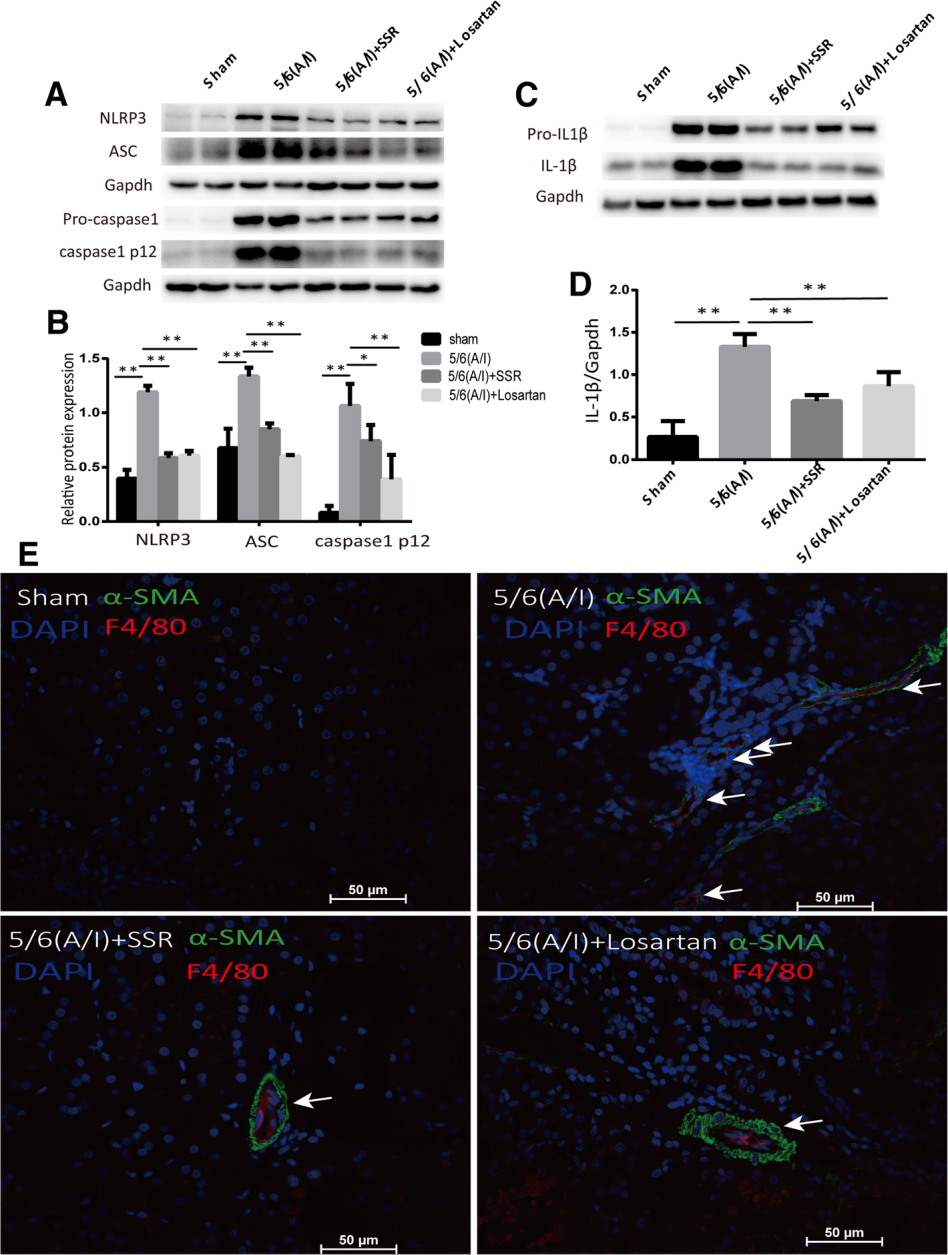
SSR inhibited NLRP3 inflammasome activation and IL-1βsecretion in 5/6(A/I) rats. (**a**) The protein expression of NLRP3,ASC, Pro-caspase1 and Caspase-1 p12 was determined by immunoblotting. (**b**) Western blot quantification of NLRP3, ASC, and Caspase-1 p12 levels. (*n* = 6) (**c**) The protein levels of Pro-IL1β and IL1βwere determined by immunoblotting. (d) The ratio of IL-1βto GAPDH protein was determined. (n = 6) (**e**) Representative images of IF staining for α-SMA and F4/80. Original magnification,× 400. Values are mean ± SD. *P < 0.05, **P < 0.01

Inflammation plays an important role in the development of renal fibrosis [24]. Renal NLRP3 inflammasome activation characterized by the elevation of NLRP3,ASC and Caspase-1 activation at protein levels was observed in the remnant kidneys with CRF,which was restored by SSR (Fig. 2(a) and2(b)).Results of inflammasome activation led to secretion of proinflammatory cytokine IL-1β(Fig. 2(c) and (d)).Consistent with above results, SSR decreased protein levels of mature IL-1β compared with 5/6(A/I) operation group,which was determined by immunoblotting for the 17-KD cytokine.

(Fig. 2(c) and (d)).

To determine whether the tubular injury and renal interstitial fibrosis are associated with inflammatory response,we mainly probed the position of F4/80 positive macrophages and myofibroblast marker α-SMA using immunofluorescence double staining. Our results presented the positional correlation between F4/80 and α-SMA (Fig. 2e). In addition,the model group reciving 5/6(A/I) operation displayed increased expression of F4/80 and α-SMA proteins compared with sham-operated group. Conversely,SSR dramatically ameliorated these expression of F4/80 and α-SMA (Fig. 2e). Taken together,our results revealed SSR effectively inhibited NLRP3/ASC/Caspase-1/IL-1β cascade and decreased inflammatory infiltration in the remnant kidneys,whcih may be associated with alleviated renal injury and interstitial fibrosis.

### SSR reduced Smad3 acetylation in 5/6(A/I) rats by regulating Sirt1/Smad3 deacetylation pathway

**Fig. 3:**
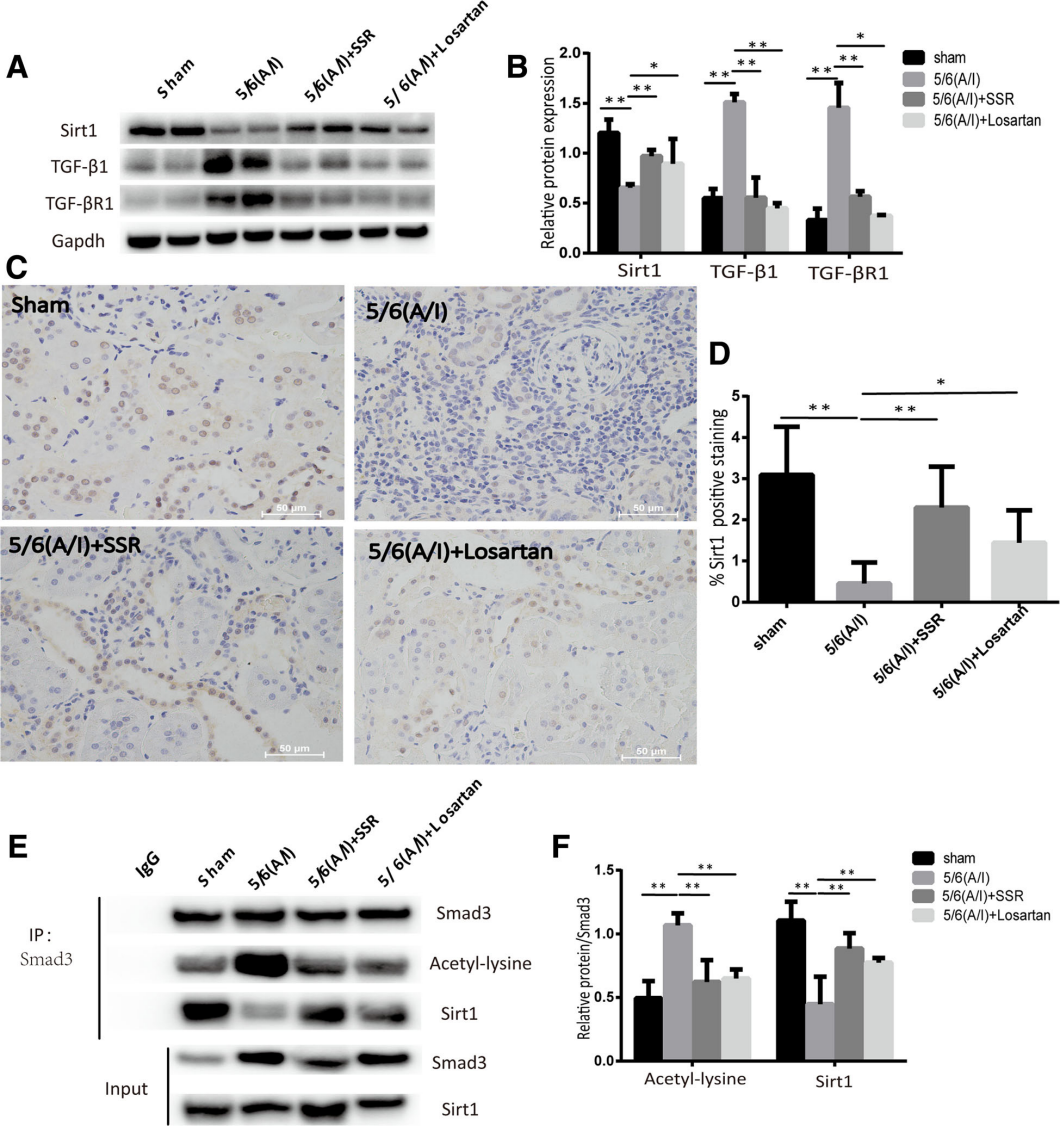
SSR reduced Smad3 acetylation in 5/6(A/I) rats by regulating Sirt1/Smad3 deacetylation pathway. (**a**) The protein levels of Sirt1,TGF-β1 and TGF-βR1 were determined by immunoblotting. (**b**) The ratio of Sirt1,TGF-β1 and TGF-βR1 to GAPDH protein was calculated. (n = 6) (**c**) Representative images of IHC staining for Sirt1. 400 × magnification. (**d**) Quantitative analysis of Sirt1 positive area(n = 4)(e) Immunoprecipitation and western blot analysis of Sirt1 and Smad3 acetylation in each group. (f) The ratio of Sirt1 and acetylated Smad3 to precipitated Smad3 protein was calculated. (*n* = 4) Values are mean ± SD. *P < 0.05, **P < 0.01

Previous reports have shown that Sirt1 could mediate a variety of cellular responses by deacetylating lysine residues such as p53, NF-κB, and PGC-1α [25–27]. In this study, we first measured the expression of Sirt1 protein. As shown in Fig. 3(a), (b), (c)and (d),compared wtih sham-operated group,immunoblotting and IHC staining showed that injury to kidney decreased the expression of Sirt1 protein,whereas treatment with SSR markedly preserved Sirt1 expression (Fig. 3(a), (b), (c)and (d)).IHC staining confirmed Sirt1 is mainly located in the nucleus (Fig. 3c).Moreover,immunoblotting examination showed expression of typeITGF-βR (TGF-βR1) and TGF-β1 proteins was increased in the 5/6 (A/I) model group as compared to that in the sham-operated group (Fig. 3(a) and (b)).Meanwhile, SSR suppressed the expression of TGF-β1 and TGF-βR1 proteins compared with model group.

In addition, to assess the levels of acetylated Smad3,Smad3 was immunoprecipitated and the acetyl group was determined by an anti-acetyl antibody. As shown in Fig. 3(e) and (f),we observed that the acetylation levels of Smad3 increased in 5/6(A/I) rats and decreased in the rats treated with SSR.Furthermore,co-immunoprecipitation and Western blot studies revealed a direct interaction between Smad3 and Sirt1.The Smad3-Sirt1 binding was reduced in model group, which could be associated with increased levels of acetylated Smad3 (Fig. 3(e) and (f)).Conversely,SSR significantly increased the binding of Sirt1 and Smad3 and reduced acetylation levels of Smad3 (Fig. 3(e) and (f)).These data strongly suggest that renal protective effect of SSR may be associated with Sirt1/Smad3 deacetylation pathway.

## Discussion

In the present study, we found that SSR could significantly suppress progressive activation of NLRP3/ASC/Caspase-1/IL-1β cascade and up-regulate Sirt1/Smad3 deacetylation pathway in the 5/6 (A/I) rat model. These changes could inhibit the expression of Col-III,the ECM protein,as well as E-cadherin,the epithelial cell marker. Together, these results identified that SSR significantly attenuated renal injury and fibrosis in the CRF model. In addition, our results showed that losartan, an angiotensin receptor blocker, seemed to be more effective than SSR in attenuating renal injury and fibrosis, inhibiting activation of NLRP3 complex and down-regulating the expression of TGF-β1 and TGF-βR1 proteins. Losartan may significantly reduce the increase of angiotensin II (Ang-II) induced by ischemia because 5/6(A/I) model is a typical intrarenal ischemia model [22, 28]. However, the specific mechanisms need further research.

Nonmicrobial (sterile) inflammatory response is an important characteristic of many CKDs [29].NLRP3 inflammasome is known to be activated by a variety of nonmicrobial signals,and therefore, NLRP3-dependent inflammation could be an attractive candidate as a mediator of the inflammatory component observed in CKD [8]. Previous studies reported NLRP3 complex was involved in the progression of kidney diseases in UUO model [9],ischemia/reperfusion injury (IRI) [30], and diabetic nephropathy [31] etc. and traditional Chinese herbs had obvious inhibition on it. Ren et al. [32]. reported Coptidis Rhizoma could attenuate early renal injury in obesity-related glomerulopathy (ORG) rats and the mechanisms were associated with the inhibition of NLRP3 inflammasome. Chang et al. [33]. demonstrated that rhein suppressed the NLRP3 inflammasome and macrophage activation in urate crystal-induced gouty inflammation. In addition, many active ingredients of SSR, such as magnesium lithospermate B from Salvia miltiorrhiza bunge, ferulic acid from Angelica sinensis,icariin from Epimedium, have shown anti-inflammatory effects in kidney diseases [34–36]. The activation of NF-κB signaling pathway stimulated renal inflammation in different models [37, 38]. Meanwhile,many reports revealed that NF-κB signaling pathway played a prime role in NLRP3 inflammasome activation [39, 40]. We have investigated the activity of NF-κB by phosphorylation of p65, its translocation to nucleus. Our results showed that SSR significantly reduced the nuclear translocation of p65 in 5/6(A/I) model [20]. These results prompted us to further study the possible role of NLRP3 inflammasome in 5/6(A/I) model, as well as the mechanisms of SSR attenuating renal fibrosis. Our results showed progressive activation of NLRP3/ASC/Caspase-1 cascade in CRF rat model,which led to the secretion of mature IL-1β pro-inflammatory factor. Inflammation initiates and sustains renal tubulointerstitial fibrosis [41],therefore,we probed colocalization of F4/80,a representative hallmark of macrophages,and α-SMA,a marker of fibroblast activation using immunofluorescence double staining. Our data showed that the increased F4/80 and α-SMA co-localized in the renal tubular areas. Conversely, activation of NLRP3 inflammasome analysed by immunoblotting and image analysis of IF staining showed significant down-regulation in treatment with SSR,verifying that one of the mechanisms by which SSR attenuates renal injury and delays the progression of renal interstitial fibrosis could be to inhibit inflammatory response induced by activation of NLRP3 complex.

Chronic inflammation is integrally associated with progressive CKD, as cytokines (TGF-β1, IL-18, IL-1β,etc.) produced by infiltrating leukocytes such as macrophages contribute significantly to renal injury and fibrosis [42].TGF-β1 promotes tissue fibrosis through the Smad3 pathway. While phosphorylation regulates Smad3 function, acetylation/de-acetylation of specific lysine residues in Smad3 has been shown to regulate TGF-β1 signaling by non-canonical signaling pathways [15]. In vitro,previous studies confirmed Smad3 could be acetylated in response to TGF-β1 stimulation [43, 44]. Recently, the lysine deacetylase sirtuin 1 (SIRT1) has been shown to exert renal protective effects [45]. Huang et al. [46]. reported that resveratrol, an activator of the Sirt1, ameliorated renal fibrosis by inhibiting the TGF-β1/Smad3 pathway. Here, we investigated whether pharmacological activation of Sirt1 would induce renal protection in 5/6(A/I) model. In this study, we showed the binding of Sirt1 with Samd3 and inhibition of this binding was associated with increased levels of acetylated Smad3. SSR down-regulated the acetylation levels of Smad3 by activating Sirt1 deacetylation pathway. These data highlight that renal Sirt1/Smad3 deacetylation pathway could be an antifibrotic factor and a potential therapeutic strategy for CKD.

## Conclusions

In summary, we demonstrated that SSR could mediate renal protection by inhibiting NLRP3/ASC/caspase-1/IL-1β pathway and, furthermore,strengthen the antifibrotic effects by up-regulating Sirt1/Smad3 deacetylation pathway in 5/6th (A/I) model rats with CRF.

## Abbreviations

5/6 (A/I): 5/6ablation/ infarction
Ang-II: AngiotensinII
BUN: Blood urea nitrogen
CKD: Chronic kidney disease
Col-I: Collagen-I
Col-III: Collagen-III
CRF: Chronic renal failure
ECM: Extracellular matrix
eGFRs: Evaluated glomerular filtration rates
FN: Fibronectin
GS: Glomerulosclerosis
IF: Immunofluorescence
IHC: Immunohistochemical
IL: Interleukin
IRI: Ischemia/reperfusion
ORG: Obesity-related glomerulopathy
PAS: Periodic acid-Schiff
Scr: Serum creatinine
SD: Sprague-Dawley
Sirt1: Sirtuin1
SSR: ShenShuai IIRecipe
TCM: Traditional Chinese medicine
TGFβ-1: Transforming growth factorβ-1
TGF-βR1: TypeITGF-βR
α-SMA: α-smooth muscle actin
